# Fosfomycin as a potential therapy for the treatment of systemic infections: a population pharmacokinetic model to simulate multiple dosing regimens

**DOI:** 10.1002/prp2.378

**Published:** 2018-02-07

**Authors:** Natalia V. Ortiz Zacarías, Anneke C. Dijkmans, Jacobus Burggraaf, Johan W. Mouton, Erik B. Wilms, Cees van Nieuwkoop, Daan J. Touw, Ingrid M. C. Kamerling, Jasper Stevens

**Affiliations:** ^1^ Centre for Human Drug Research Leiden the Netherlands; ^2^ Department of Medical Microbiology Medical Center Haaglanden The Hague the Netherlands; ^3^ Department of Medical Microbiology Radboud University Nijmegen Medical Center Nijmegen the Netherlands; ^4^ Department of Microbiology and Infectious Diseases Erasmus MC Rotterdam the Netherlands; ^5^ Hospital Pharmacy The Hague Hospitals The Hague the Netherlands; ^6^ Department of Internal Medicine The Hague Hospitals The Hague the Netherlands; ^7^ University Groningen University Medical Center Groningen Department of Clinical Pharmacy and Pharmacology Groningen the Netherlands

**Keywords:** fosfomycin, fosfomycin tromethamine, multi‐drug resistant, population pharmacokinetics, simulation, systemic infections

## Abstract

Fosfomycin has emerged as a potential therapy for multidrug‐resistant bacterial infections. In most European countries, the oral formulation is only approved as a 3 g single dose for treatment of uncomplicated cystitis. However, for the treatment of complicated systemic infections, this dose regimen is unlikely to reach efficacious serum and tissue concentrations. This study aims to investigate different fosfomycin‐dosing regimens to evaluate its rationale for treatment of systemic infections. Serum concentration‐time profiles of fosfomycin were simulated using a population pharmacokinetic model based on published pharmacokinetic parameter values, their uncertainty, inter‐individual variability and covariates. The model was validated on published data and used to simulate a wide range of dosing regimens for oral and intravenous administration of fosfomycin. Finally, based on the minimum inhibitory concentration for *E. coli*, surrogate pharmacodynamic indices were calculated for each dosing regimen. This is the first population pharmacokinetic model to describe the oral pharmacokinetics of fosfomycin using data from different literature sources. The model and surrogate pharmacodynamic indices provide quantitative evidence that a dosing regimen of 6–12 g per day divided in 3 doses is required to obtain efficacious exposure and may serve as a first step in the treatment of systemic multi‐drug‐resistant bacterial infections.

**Abbreviation**
AUC: area under the concentration‐time curve
CI: confidence intervals
CL: clearance
ESBL: extended‐spectrum beta‐lactamases
GI: gastrointestinal
MBL: metallo‐β‐lactamases
MDR: multi‐drug resistant
MIC: minimum inhibitory concentration
PI: prediction interval
PK: pharmacokinetic
UTIs: urinary tract infections

## 1 INTRODUCTION

Antibacterial resistance remains one of the major threats to human health, despite its identification as one of the worldwide priority conditions by the WHO over a decade ago.^1^, ^2^, ^3^ Particularly alarming is the rise in number and spread of multi‐drug resistant (MDR) bacterial strains and a poor pipeline of new Gram‐negative antibiotics.^4^, ^5^, ^6^, ^7^

To battle MDR bacteria strains, the reassessment and reintroduction of ‘old’ antibiotics have emerged as alternative solution to circumvent the long and costly process of developing new antibiotics.^8^, ^9^ One of such ‘old’ antibiotics is fosfomycin, developed more than 40 years ago.^10^ Fosfomycin is a broad spectrum antibiotic which exerts its bactericidal activity by irreversibly inhibiting the early stages of the bacterial cell wall synthesis.^11^

MDR Gram‐negative bacteria are responsible for around two‐thirds of the deaths by MDR‐bacterial infections in Europe.^6^ Fosfomycin exhibits in vitro and in vivo antibacterial activity against a wide range of both Gram‐positive and Gram‐negative bacteria, including several MDR‐strains.^12^, ^13^, ^14^, ^15^, ^16^, ^17^ Even most of the extensively drug‐resistant (XDR) Enterobacteriaceae strains still remain susceptible to fosfomycin, including those expressing extended‐spectrum beta‐lactamases (ESBL) or metallo‐β‐lactamases (MBL).^14^, ^15^, ^16^, ^18^ In addition, fosfomycin has been suggested as add‐on therapy for infections caused by MDR‐*P. aeruginosa,* one of the main pathogens associated with nosocomial‐acquired infections.^16^, ^17^, ^19^

Fosfomycin has been marketed in different formulations including fosfomycin tromethamine for oral administration and fosfomycin disodium for intravenous administration.^20^ In most European countries, only the oral formulation is available and approved as a single 3 g dose for the treatment of uncomplicated urinary tract infections (UTIs) in women. This single‐dose regimen is not efficacious for the treatment of systemic MDR bacterial infections, making the prospective evaluation of new oral dosing regimens a necessity. A multiple‐dose regimen of oral fosfomycin tromethamine has been proposed for the treatment of complicated UTIs, including those due to MDR‐bacteria.^21^, ^22^ However, more studies are urgently needed to determine the optimal oral dose regimen to achieve efficacious systemic exposure.

Few pharmacokinetic (PK) models for fosfomycin have been described in literature, which were developed on different study designs, limited numbers of subjects and different model structures.^23^, ^24^, ^25^, ^26^ PK modeling techniques allow integration of different study designs, on the basis that despite study differences the underlying *population* pharmacokinetics are similar, as commonly applied in dose‐regimen selection.^27^

To assess the feasibility of a multiple oral‐dose regimen with fosfomycin tromethamine for systemic infections, a combined PK model for intravenous and oral administration was built on PK parameters reported in literature in order to simulate various serum‐concentration time profiles. In addition, surrogate pharmacodynamic indices were calculated, based on the minimum inhibitory concentration (MIC) representing the epidemiological cut‐off value for *E. coli*,^28^ to estimate its clinical efficacy.

## 2 METHODS

### 2.1 PK model

The structural model for intravenous administration was based on a previously reported two‐compartment population PK model of fosfomycin, developed on 12 patients scheduled for abscess drainage.^25^ The model was parameterized in terms of elimination rate constant (*k*_e_), volumes of distribution for the central (*V*c) and peripheral compartments (*V*p) and intercompartmental clearance (Q). The rate and duration of infusion were parameterized by Q_inf_ and *t*_inf_, respectively.

**Figure 1:**
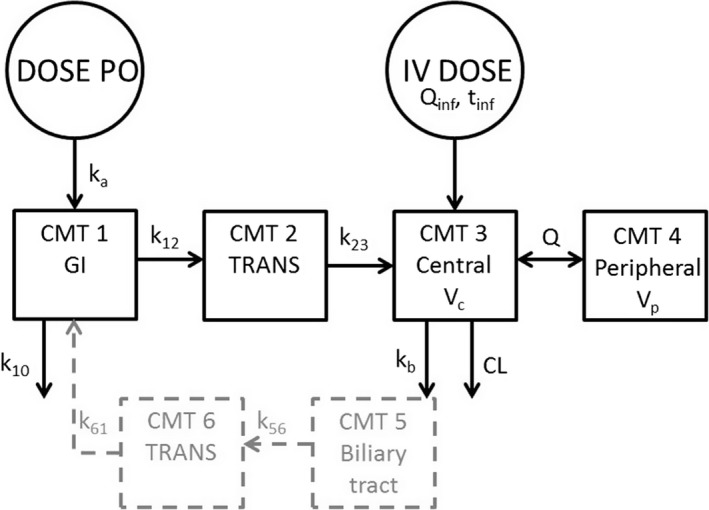
The two compartment PK model structure used for the simulations of fosfomycin multiple‐dose regimens (black), together with the excluded enterohepatic recirculation (gray). CL, clearance; CMT, compartment with associated number; k10, the first‐order loss prior to reaching CMT 2; k12, k23, k56, k61, rate constants between compartments; kb, biliary elimination; GI; gastrointestinal; Q, intercompartmental clearance; Q_inf_ infusion rate constant; t_inf_, infusion time; TRANS, transit; *V*c, central volume of distribution; *V*p, peripheral volume of distribution

To include oral administration of fosfomycin tromethamine, the model was extended with a gastrointestinal‐ (GI) and a transit component (TRANS), based on a PK model published by Segre et al., that was developed after oral and intravenous administration in 5 healthy volunteers.^24^ This model was parameterized in terms of rate constants *k*_*ij*_, representing the different rates of drug transfer from the *i* ^*th*^ compartment to the *j* ^*th*^ compartment, including a k_10_, representing the first order loss of dose, hence correcting for oral bioavailability. Additionally, a transfer constant representing biliary clearance of the drug (*k*_*b*_) was included in the oral PK model. As literature is inconclusive on reabsorption of fosfomycin,^24^, ^29^, ^30^ models with and without enterohepatic recirculation were compared to published data in order to evaluate its descriptive impact on the simulations. The PK model structures used for the simulations of different multiple‐dose regimens after intravenous and oral administration of fosfomycin are presented in Figure 1.

Individual PK parameters were simulated according to Equation (1).

(1) $$\theta_{i}=\theta_{\mathrm{TV}}*\exp\left(\eta_{i}\right),$$

where θ_*i*_ is the PK parameter for the *i* ^*th*^ individual, θ_*TV*_ the typical population PK parameter, and η_*i*_ the interindividual variability (IIV) for the *i* ^*th*^ individual.Here, IIV was reported to be log‐normally distributed for CL, *V*c, and *V*p**,** 25 and incorporated as such in the model; η is assumed to be normally distributed around 0 with its reported variance ω^*2*^.

The θ_*TV*_ is simulated based on literature values of mean population PK parameters (θ_*p*_) and their uncertainty in terms of variance [based on reported standard deviation (SD) and/or 90% confidence intervals (CI)], thus resulting in an uncertainty distribution of the population PK parameter. Both θ_*p*_ and its variance were log‐transformed to avoid negative values, according to Equation (2) and Equation (3).^31^

(2) $$\theta_{p,LN}=ln\theta_{p,N}-\frac{1}{2}\omega_{\mathrm{LN}}^{2}$$

(3) $$\omega_{\mathrm{LN}}^{2}=ln\left(\frac{\sigma_{N}^{2}}{\theta_{p,N}^{2}}+1\right),$$

where subscript _*LN*_ refers to the log domain, and _*N*_ refers to the normal domain. Subsequently, θ_*TV*_ was calculated according to Equation 4.

(4) $$\theta_{\mathrm{TV}}=exp\left(\theta_{p,LN}+\omega_{\mathrm{LN}}^{2}\right)$$

### 2.2 Covariates

A mean‐centered linear relationship between creatinine clearance (CL_CR_) and clearance (CL) was reported,^25^ and incorporated as such in the simulated clearance for the *i* ^*th*^ individual (*CL*_*i*_, Equation (5)).

(5) $$CL_{i}=\left(CL_{\mathrm{TV}}+\left(0.0141*(CL_{CR,i}-103)\right)\right)*\exp\left(\eta_{i}\right),$$

where *CL*_*TV*_ is the literature derived mean population parameter with its uncertainty (Equation 4), *CL*_*CR,i*_ is the creatinine clearance and η_*i*_ the IIV for the *i* ^*th*^ individual. The *CL*_*CR,i*_ and normalization factor (103) were obtained from Sauermann et al.^32^ To simulate *CL*_*CR,i*_, samples were drawn from a distribution with a mean of 103 and standard deviation 41, which was limited between the minimal and maximal reported values.^32^

### 2.3 Simulations

One thousand (1000) individual PK parameter sets (θ_*i*_) were randomly sampled using the distributions for parameter uncertainty and IIV, with resampling. The resulting individual PK parameter sets were then used to simulate individual plasma fosfomycin concentrations over time. The mean PK parameters, uncertainty and IIV used for the simulations are listed in Table 1. All simulations were performed in R (version 2.13.1 33) using the lsoda (deSolve Package 1.10‐3) and mvrnorm functions (MASS Package v7.3‐8), within the RStudio^34^ interface (version 0.98.501).

**Table 1:** Pharmacokinetic parameter values used in the simulations

Parameter	Mean estimate (90% CI or ±SD)	IIV	Uncertainty (variance)^a^	Reference
CL (L/h)^b^	5.808 (3.792–7.80)	0.238	1.4841	Kjellsson et al.^25^
*V*c (L)	10.1 (5.36–14.8)	0.238*1.64	8.2329	Kjellsson et al.^25^
*V*p (L)	9.80 (5.70–13.9)	0.197	6.2120	Kjellsson et al.^25^
Q (L/h)^b^	15.36 (9.12–21.6)	NI	14.3892	Kjellsson et al.^25^
COV_CLCR‐CL_	0.0141	–	–	
k_10_ (h^−1^)	1.24 ± 0.55	ND	0.3025	Segre et al.^24^
k_12_ (h^−1^)	1.69 ± 0.62	ND	0.3844	Segre et al.^24^
k_23_ (h^−1^)	0.34 ± 0.10	ND	0.0100	Segre et al.^24^
k_b_ (h^−1^)	0.50 ± 0.18	ND	0.0324	Segre et al.^24^

CL, clearance; *V*c, volume of distribution of central compartment; *V*p, volume of distribution of peripheral compartment; Q, intercompartmental clearance; COV_CLCR‐CL_^,^ linear relationship between creatinine clearance and CL; *k*_*x*,*y*_, rate constants from compartment *x* to *y*; NI, not identified; k_b_, rate constant biliary elimination; ND, no data available.

a Calculated from the 90% CI or SD.

b Value converted to match units.

### 2.4 Model validation

The validation of the PK models was performed by simulating previously published study designs and visually comparing the 90% prediction interval (PI) of the simulations to the observed data reported in literature. In short, the previously published study designs in healthy volunteers were, for intravenous administration, 8 doses of 500 mg every 6 hours^35^; 500 mg in 5 min infusion^23^; and 50 mg/kg bolus.^24^ For single‐dose oral administration, dosing regimens were 50 mg/kg, 2 g and 5 g.^24^

### 2.5 Alternative dosing regimens and calculation of PK/PD indices

Once validated, the different oral dosing regimens were simulated to assess the feasibility of a multiple dosing regimen. These scenarios included the simulation of total daily doses ranging from 3 g to 45 g once or divided into two or three times per day for oral fosfomycin tromethamine.

PK parameters were obtained in R and included: maximum serum concentration (C_max_), time to reach C_max_ (T_max_), area under the serum concentration–time curve (linear trapezoidal rule with 0.1 h time‐steps) over the dosing interval (AUC_0‐tau_), and AUC from time 0 to time of the last simulated concentration (AUC_0‐last_).

Surrogate pharmacodynamic indices were based on the minimum inhibitory concentration (MIC) of 8 mg/L, as this represents the epidemiological cut‐off value for *E. coli* according to EUCAST 28 and include: C_max_/MIC, AUC/MIC, time above MIC (T_>MIC_) and percentage of T_>MIC_ during the dose interval (%T_>MIC_). Primarily, the mean estimated values of C_max_ and AUC during 24 hour at steady state were used. The C_max_/MIC and %T_>MIC_ were calculated over the length of a dose interval at steady state, while AUC/MIC was calculated over a period of 24 hours at steady state as defined by Mouton et al.^36^ Secondly, the lower 90% prediction interval (PI) of the simulated plasma concentration‐time profiles was used, e.g., 95% of all subjects will have higher exposure compared to this PI.

## 3 RESULTS

### 3.1 PK Models

The contribution of enterohepatic recirculation on improvement of descriptive properties of the model proved to be marginal; the median concentrations and 90% PI did not differ substantially. The slight changes were considered to be of no clinical relevance. Secondly, as there is also no consistent proof for enterohepatic recirculation in literature, it was decided to exclude this PK property from the model. The parameter *k*_*b*_ was kept in the model as this rate constant for apparent biliary elimination is required to attest for the total elimination of fosfomycin.

**Figure 2:**
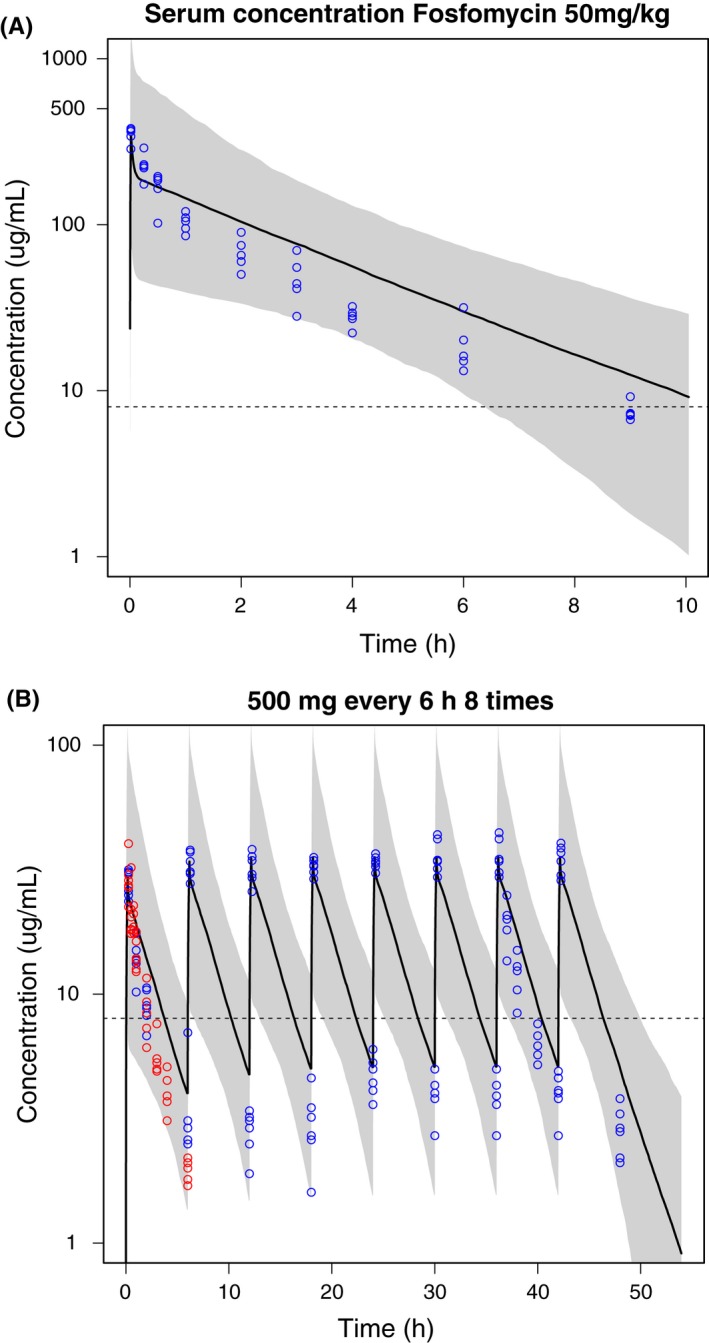
Mean plasma fosfomycin concentration‐time profiles (black line) and 90% prediction interval (gray area) of 1000 simulated subjects with observations (circles): (A) simulations and data after 1 minute iv bolus injection of 50 mg/kg fosfomycin disodium^24^; (B) simulations after 500 mg of fosfomycin disodium in a 5‐10 minute short iv infusion with data (blue; data obtained by Kwan et al.,^23^ red; data obtained by Cadorniga^35^). The dashed line represents the minimum inhibitory concentration

**Figure 3:**
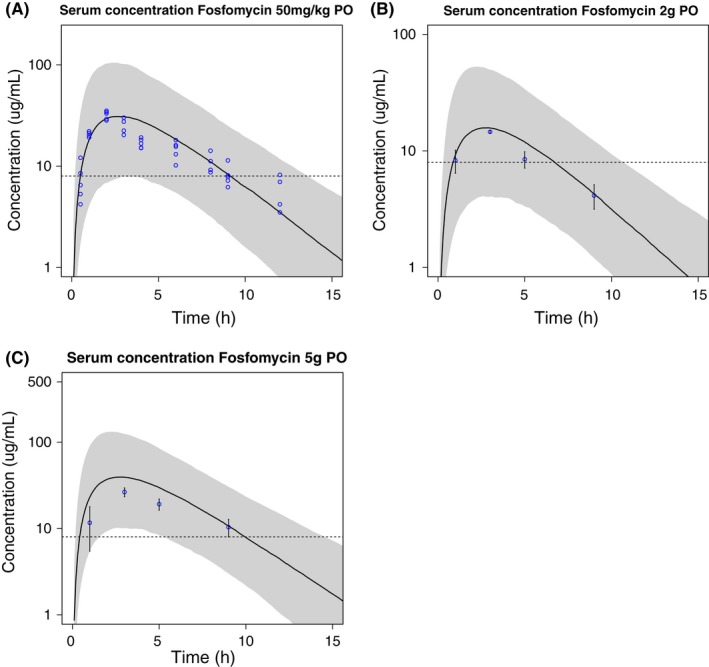
Mean serum fosfomycin concentration‐time profiles (black line) and 90% prediction interval (gray area) of 1000 simulated subjects with reported observations^24^ after oral administration of fosfomycin tromethamine: (A) 50 mg/kg with data (blue circles,^24^ (B) 2 g with reported mean values ± SD and (C) 5 g with reported mean values ± SD. The dashed line represents the minimum inhibitory concentration

All observations following intravenous (Figure 2) and oral dosing (Figure 3) lie within the 90% PI of the PK model. For the intravenous simulations, C_max_ is well described and the median slope of the terminal elimination phase follows the slope of the data. However, the terminal elimination phase and trough concentrations seem overpredicted by the model. Following the multiple 500 mg dose in 8 hours dosing intervals, no accumulation occurs and the simulated median concentration remains above the MIC until approximately 5 hours after dosing. For the oral simulations, the median C_max_ seems well predicted although the shape of the concentration‐time curve in the terminal phase seems steeper compared to the data. Following the single 50 mg/kg dose, the simulated median serum concentration remains above the MIC until approximately 10 hours after dosing. As all data points lie within the 90% PI of the simulations, the PI is wider than expected based on the data, indicating that the variability of the model is overestimated.

### 3.2 Simulation of different multiple‐dose regimens and calculation of PK/PD Indices

**Figure 4:**
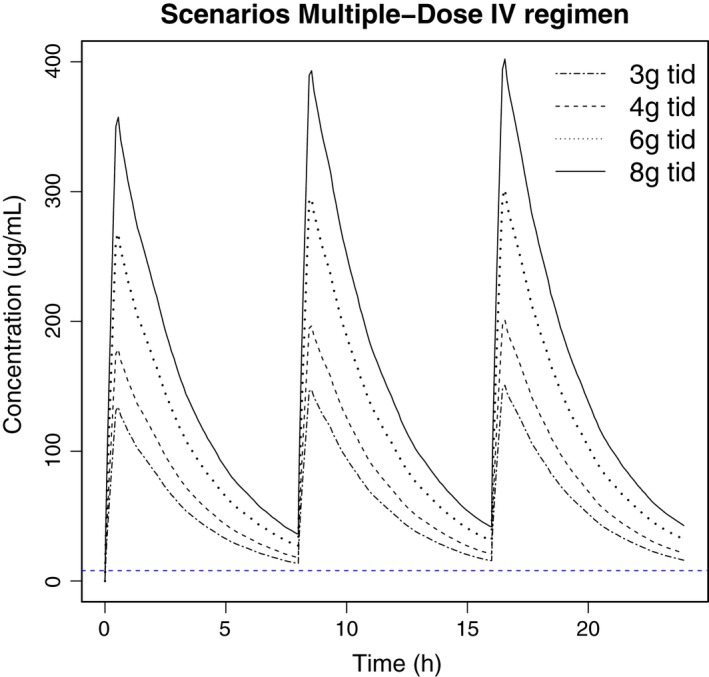
Median serum fosfomycin concentration‐time profiles of 1000 simulated subjects after three times daily (tid) iv bolus dosing of 3, 4, 6 and 8 mg fosfomycindisodium. Horizontal dashed line represents the minimum inhibitory concentration

Different multiple‐dose regimens after oral administration of fosfomycin were simulated using the validated PK model. Figure 4 shows the medians of the predicted PK profiles of 1000 subjects after intravenous administration of 3, 4, 6, or 8 g of fosfomycin every 8 hours by 30 min infusion, as well as the MIC. In addition, simulation of different dosing schedules such as 4 g and 6 g every 6 hours were also conducted (data not shown). All simulated intravenous regimens reached serum concentrations above the MIC. The surrogate pharmacodynamic indices and mean PK measures for each dosing regimen are shown in Table 2. All intravenous dosing regimens simulated produced C_max_ levels of at least 18‐fold over the MIC, AUC/MIC values from 180 to 500, and a 100%T_>MIC_.

**Table 2:** Mean surrogate pharmacodynamic indices for different intravenous dosing regimens of fosfomycin disodium, using a MIC of 8 mg/L

Dose (g)	Interval (h)	C_max_ (mg/L)	C_max_/MIC	AUC (mg/L*h)	AUC/MIC8	%T_>MIC_
3	8	151.41	18.93	1490.82	186.35	100
4	8	201.88	25.24	1987.76	248.47	100
4	6	224.04	28.00	2684.44	335.55	100
6	8	302.83	37.85	2981.64	372.70	100
6	6	336.05	42.01	4026.66	503.33	100
8	8	403.77	50.47	3975.52	496.94	100

C_max_, maximum concentration; MIC, minimum inhibitory concentration; AUC, area under the concentration‐time curve; %T_>MIC_, time above the MIC during a dose interval, expressed as percentage.

**Figure 5:**
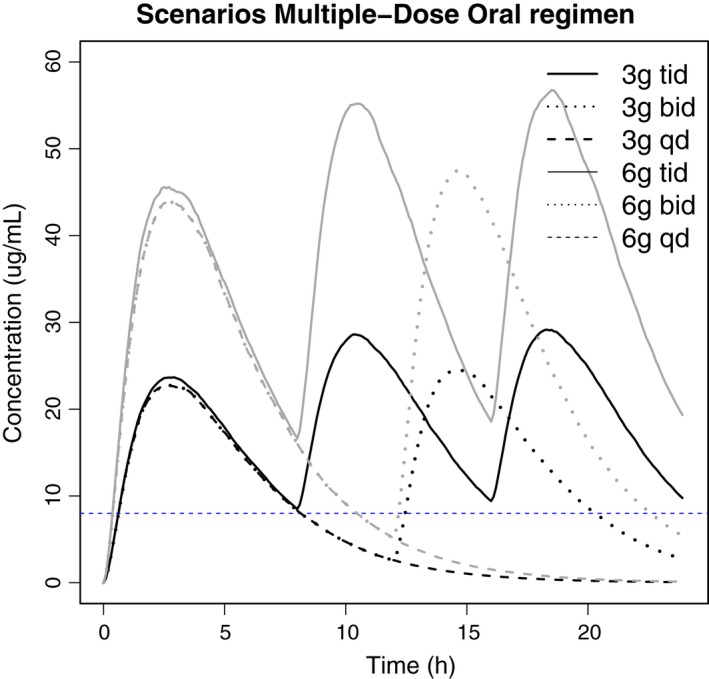
Median serum concentration‐time profiles of fosfomycin simulated in 1000 subjects following oral administration of 3 or 6 g of fosfomycintromethamine with various dose regimens: single dose (sd), two times daily (bid) or three times daily (tid). Dashed blue line represents the minimum inhibitory concentration of 8 mg/l

Several oral dose regimens were simulated for doses of 3 g and 6 g of fosfomycin tromethamine, including a single dose per day (qd), two times daily (bid) and three times daily (tid). The predicted medians of these different dose regimens as presented in Figure 5 show that the medians of all first doses reached serum concentrations above the MIC. For both dose groups, concentrations only maintain above the MIC for the entire duration of the day following tid dosing. As shown in Table 3, a 2 g tid dose would also not suffice to reach a %T_>MIC_ of 100%. Interestingly, the currently clinically approved 3 g single oral dose for UTIs may achieve efficacious concentrations in urine, however, it only achieves a %T_>MIC_ of around 30% in serum. Although most of the regimens reached a high %T_>MIC_, comparable to the intravenous regimens, the C_max_/MIC and AUC/MIC values are lower than those in intravenous regimens: the C_max/MIC_ is 17.78 after 15 mg bid and the AUC/MIC values range from 37 to 300. Table 3 also represents the pharmacodynamic indices based on the lower 90% PI of the plasma concentration‐time simulations. These results show that for some individuals, a minimum dose of 4 g tid will be required in order to reach a C_max_ that exceeds the MIC, and remains above the MIC for more than 50% of the dose interval.

**Table 3:** Surrogate pharmacodynamic indices based on the median (med) and lower limit of the 90% prediction interval (90PI) PK simulations for different oral dosing regimens of fosfomycin tromethamine, using a MIC of 8 mg/L

Dose(g)	Interval(h)	C_max_ (mg/L) med/90PI	C_max_/MIC med/90PI	AUC (mg/L*h) med/90PI	AUC/MIC med/90PI	%T_>MIC_ med/90PI
2	8	18.96/5.16	2.37/0.65	316.95/92.18	39.62/11.52	84/0
3	8	28.44/7.75	3.56/0.97	475.42/138.26	59.43/17.28	100/0
3	12	24.52/6.60	3.07/0.82	313.48/88.52	39.19/11.06	66/0
3	24	22.87/6.05	2.86/0.76	154.26/41.58	19.28/5.20	31/0
4	8	37.93/10.33	4.74/1.29	633.89/184.35	79.24/23.04	100/51.57
5	8	47.41/12.91	5.93/1.61	792.36/230.44	99.05/28.80	100/67.63
6	8	56.89/15.50	7.11/1.94	950.84/276.53	118.85/34.57	100/78.75
6	12	47.70/13.34	5.96/1.67	602.87/178.67	75.36/22.33	87/45.76
6	24	44.12/12.12	5.51/1.52	296.83/83.11	37.10/10.39	42/20.44
7	8	66.37	8.30	1109.31	138.66	100
8	8	75.85	9.48	1267.78	158.47	100
9	8	85.33	10.67	1426.26	178.28	100
10	8	94.81	11.85	1584.73	198.09	100
11	8	104.30	13.04	1743.20	217.90	100
12	8	113.78	14.22	1901.67	237.71	100
15	8	142.22	17.78	2377.09	297.14	100

C_max_, maximum concentration; MIC, minimum inhibitory concentration; AUC, area under the concentration–time curve; %T_>MIC_, time above the MIC during a dose interval, expressed as percentage.

## 4 DISCUSSION

This is the first population PK model to describe the oral pharmacokinetics of fosfomycin, using data from different literature sources. The study provides quantitative evidence that an oral dosing regimen of 6–12 g per day divided in 3 doses is required to obtain serum concentrations above the MIC for at least 50% of the dose interval. This may serve as a first step in the treatment of systemic infections by MDR bacteria with a similar MIC compared to *E.coli*.

Model validation showed a slight bias in the description of literature data and overprediction of variability. The slight bias can be explained by the use of few subjects in the development of the literature models causing relatively high parameter uncertainty and IIV, which accumulates in large prediction intervals. Following intravenous simulation, late PK time points seem overestimated while for oral simulations time points after 15 hours seem underestimated, which may lead to bias in accumulation following multiple dosing regimens. In general, the reported population PK parameters used in our simulations were within the CI reported in a recent publication on intravenous fosfomycin infusion in critically ill patients. Compared to the volume of distribution in our simulations, the publication reports a relatively high volume of distribution, which the authors attest to pathophysiological changes in their critically ill patient population.^26^ We acknowledge the quantitative and qualitative lack of data in literature, which is the case for many drugs that have been developed in the past. For this reason, we stress the importance of additional clinical data to ascertain whether oral fosfomycin may be used for the treatment of systemic.

The suggested daily oral doses of fosfomycin tromethamine to achieve an effective serum concentration exceed the currently approved single dose of 3 g. To our knowledge, safety and tolerability has not been investigated in vivo, using higher oral doses. Alternative approaches to avoid such higher doses when dealing with systemic MDR infections may lie in synergistic combinations with other antibiotics, such as imipenem for treatment of methicillin‐resistent *Staphylococcus aureus*,^37^ or approval of intravenous fosfomycin formulations. Yet, more studies are urgently needed to assess the PK, safety, tolerability, and efficacy of fosfomycin in multiple‐dose regimens and synergistic combinations.

The broad range of daily doses suggested with these simulations (from 6 up to 12 g per day) can be explained, in part, by the relatively large parameter uncertainty and IIV reported in literature. To our knowledge, serum creatinine clearance is the only reported covariate in literature that explains part of the IIV. In addition, disease state may explain IIV of volume of distribution.^26^ These aspects contribute to wide prediction intervals around the means of the simulations. An effect of bodyweight on volume of distribution has been used in a study but was not statistically supported.^26^ Inclusion of more data and demographics would reduce the parameter uncertainty and improve quantitation of the IIV and is anticipated to provide a more precise prediction interval. With the current available literature data, the current dosing results based on the lower 95% prediction interval may prove to be a relatively conservative approach.

In this study, different surrogate pharmacodynamic indices were used to evaluate the effect of different dose regimens on the epidemiological cut‐off value for *E. coli*. However, an important limitation in the evaluation of different dose regimens and optimization of therapy is the lack of information regarding the PD properties of fosfomycin. Few studies have attempted to characterize the PD properties of fosfomycin, but results are conflicting. Some studies pointed to a time‐dependent bactericidal activity,^38^, ^39^ while others have suggested a concentration‐dependent bactericidal activity.^40^ This again stresses the need for more data.

The lack of PD data has also affected the clinical and PD breakpoints for MDR‐bacterial infections from a regulatory perspective. In the case of fosfomycin tromethamine, the EUCAST has established clinical breakpoints for Enterobacteriaceae (Susceptible ≤32 mg/L and Resistant > 32 mg/L) which are only applicable to uncomplicated UTIs caused by Enterobacteriaceae, using a single dose of 3 g.^28^ As clinical breakpoints depend on the clinical features of the disease and the dose regimen, we chose the epidemiological cut‐off value of fosfomycin for *E. coli* to calculate the PD indices. This value is independent of the dose regimens and exclusively determined by the MIC values distribution and therefore not used to advise on clinical therapy.^41^ In this regard, further studies are urgently needed to establish the PK–PD relationships of fosfomycin. Microbiological susceptibility information could also be included in Monte Carlo simulations in order to define oral dosing regimens based on potential PK/PD targets with high probability of microbiological cure. This has been recently reported following intravenous infusion of fosfomycin in the treatment of *Klebsiella pneumoniae*,^42^ and *Pseudomonas aeruginosa*.^43^

Literature review on fosfomycin PK and simulations clearly indicate the need for further clinical research to characterize the PK and PD properties of fosfomycin tromethamine. Previous studies reported potential decreased absorption at higher doses 24, 44 and fosfomycin recirculation.^24^ In the model building, these concepts were considered but did not improve the descriptive properties of the model with regards to the available data. Also, when administering doses that are higher than the current recommended dose in the clinic, this may result in nonlinear PK.^24^, ^44^ Hence, in the design of a future clinical trial, dose regimens as well as sampling times should be chosen to optimally address these potential PK characteristics. Characterization of these processes is the key to the design of optimal multiple‐dose strategies, as saturable absorption or elimination can limit the use of higher doses and recirculation can lead to clinically relevant accumulation.

Simulations and PD indices show that a total daily oral dose of at least 6–12 g of fosfomycin tromethamine are required to achieve a therapeutic concentration to treat systemic infections, based on the epidemiological cut‐off value for *E. coli*. In light of the reported simulations, the population PK model can be used to optimize a new clinical trial to assess the PK, safety, and tolerability of fosfomycin tromethamine in multiple‐dose regimens.
